# Patterns, Receptors, and Signals: Regulation of Phagosome Maturation

**DOI:** 10.1016/j.it.2017.03.006

**Published:** 2017-06

**Authors:** Anne-Marie Pauwels, Matthias Trost, Rudi Beyaert, Eik Hoffmann

**Affiliations:** 1 Unit of Molecular Signal Transduction in Inflammation, VIB Center for Inflammation Research, Ghent, Belgium; 2 Department of Biomedical Molecular Biology, Ghent University, Ghent, Belgium; 3 MRC Protein Phosphorylation Unit, University of Dundee, Dundee, UK; 4 Institute for Cell and Molecular Biosciences, Newcastle University, Newcastle-upon-Tyne, UK; 5 Current address: Univ. Lille, CNRS, Inserm, CHU Lille, Institut Pasteur de Lille, U1019 - UMR8204 - CIIL - Center for Infection and Immunity of Lille, F-59000 Lille, France

**Keywords:** phagocytosis, phagosome maturation, inflammation, immune response, antigen presentation, host–pathogen interaction

## Abstract

Recognition of microbial pathogens and dead cells and their phagocytic uptake by specialized immune cells are essential to maintain host homeostasis. Phagosomes undergo fusion and fission events with endosomal and lysosomal compartments, a process called ‘phagosome maturation’, which leads to the degradation of the phagosomal content. However, many phagocytic cells also act as antigen-presenting cells and must balance degradation and peptide preservation. Emerging evidence indicates that receptor engagement by phagosomal cargo, as well as inflammatory mediators and cellular activation affect many aspects of phagosome maturation. Unsurprisingly, pathogens have developed strategies to hijack this machinery, thereby interfering with host immunity. Here, we highlight progress in this field, summarize findings on the impact of immune signals, and discuss consequences for pathogen elimination.

## Trends

Self and non-self immune signals are able to delay or accelerate phagosome maturation, and their effects are dependent on the phagocytic cell type, duration of stimulation, and whether the stimulus is particle bound or present in the cellular environment.

Acceleration of phagosome maturation enhances pathogen killing, while a delay in phagosome maturation preserves antigenic peptides for presentation to T cells and to initiate adaptive immune responses.

Besides its functions in pathogen killing and antigen presentation, the phagosome also functions as a signaling platform and interacts with other cell organelles.

Some pathogens are able to arrest phagosome maturation to enhance their intraphagosomal survival and replication or to promote phagosomal escape.

The latex bead phagocytosis model system combined with mass spectrometry is a powerful technique to analyze changes in the phagosomal proteome.

## Phagosome Maturation in the Context of Inflammation and Infection

Phagocytosis appeared during the evolution of unicellular eukaryotic organisms and describes the ingestion of large particles (≥0.5 μm) [1]. In Protozoa, such as *Dictyostelium discoideum*, phagocytosis serves mainly in the uptake of nutrients [2]. By contrast, phagocytes in Metazoa mostly contribute to the maintenance of homeostasis by clearing cell debris and dead cells. Furthermore, phagocytosis is an essential defense mechanism of innate immunity, by recognizing, engulfing and destroying invading microbes. Professional phagocytes, such as neutrophils, dendritic cells (DCs), monocytes, and macrophages (MΦs), have different phagocytic capacities 3, 4. However, epithelial cells, fibroblasts, and certain B lymphocyte subsets also engage in phagocytosis 5, 6, 7.

The phagocytic process is initiated by the recognition of a particle ligand by cell surface receptors. To achieve selective uptake of baits, professional phagocytes express specific but partially redundant arrays of receptors. Receptor recognition launches signaling pathways that induce remodeling of the actin cytoskeleton and extension of membrane protrusions that surround the particle to form a phagocytic cup. Once the phagocytic cup is sealed, a phagosome is formed that gradually matures via fusion and fission events with vesicles of the endocytic compartment. Initially, the formed phagosome interacts with different types of endosomes to gradually mature from an early phagosome into a late phagosome (Box 1). Endosomal fusion events with phagosomes resemble often a ‘kiss and run’ mechanism, describing transient interactions between compartments with limited exchange of contents and membranes [8], although complete fusion between these organelles can also occur. Ultimately, phagosomal fusion with lysosomal compartments is responsible for the development of a ‘phagolysosome’.

**Figure I:**
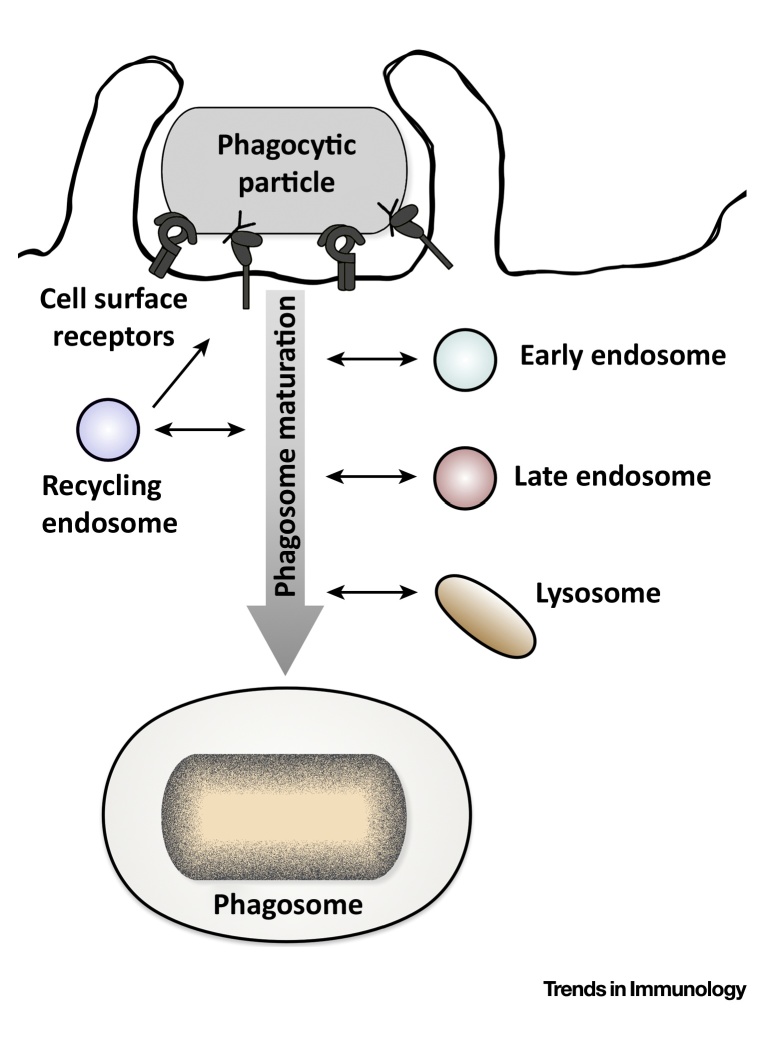
Title.

Box 1 **Molecular Details of Phagosome Maturation.**

During phagosome maturation, the phagosome interacts with endosomes and lysosomes (Figure I), which changes the phagosomal protein composition and increases its degradative capacity and antimicrobial activity over time. Here, we discuss briefly key molecules involved in phagosome maturation, which are comprehensively reviewed elsewhere [3].

Soon after engulfment, the formed phagosome fuses with early endosomes and acquires the small GTPase Rab5. The Rab5 effector Rabaptin-5 recruits the class III phosphoinositide 3-kinase vacuolar protein-sorting 34 (Vps34) [83]. The activity of Vps34 and other molecules results in the cyclic accumulation of PI3P on the phagosome, which is essential for the progression of phagosome maturation [84]. PI3P mediates the recruitment of early endosomal antigen 1 (EEA1) and the class C core vacuole/endosome tether (CORVET) complex to the phagosomal membrane and its fusion with target membranes 85, 86. In addition, the phagosome acquires the v-ATPase to initiate acidification [10].

The transition from an early phagosome to a late phagosome is marked by the conversion from Rab5 to Rab7, which induces the CORVET-to-homotypic fusion and vacuole-sorting (HOPS) switch [87]. Rab7 activity is a prerequisite for the centripetal movement of phagosomes and, therefore, is essential for further phagosome maturation [88]. In addition, the phagosome acquires LAMP-1 and LAMP-2, which are required for phagolysosomal fusion [89].

Finally, the phagosome interacts with lysosomes to develop into a phagolysosome, which is mediated by different soluble NSF attachment protein receptors (SNAREs) [90]. At this stage, the degradative capacity and antimicrobial activity of the phagosome is augmented by the acquisition of hydrolytic enzymes, such as cathepsins [91], and the production of oxygen radicals by NADPH oxidases [92].

During phagosome maturation, intraluminal vesicles are also formed for the degradation of transmembrane proteins [93]. Alternatively, certain phagosomal cargo proteins are recycled back to the plasma membrane or the *trans*-Golgi Network, mediated by different Rab proteins (Rab4, Rab11, and Rab10) and the retromer complex, respectively [94]. In DCs, phagosomes interact with the ER–Golgi intermediate compartment and the endocytic recycling compartment to enhance cross-presentation of phagosomal antigens. These interactions are mediated by different Rab proteins (Rab11a and Rab22) and SNARE proteins (Sec22b and SNAP23) 95, 96, 97.

Alt-text: Box 1

Small GTPases of the Rab family represent an important group of proteins involved in phagosome maturation. The binding of specific Rab proteins to intracellular organelles enables targeting specificity and facilitates interactions of phagosomes with different compartments over time (Box 1). Rab proteins are involved in vesicular trafficking between cell organelles by modulating the recruitment of binding partners and interactions with the cytoskeleton [9]. The **vacuolar ATPase** (v-ATPase; see Glossary) is recruited early to phagosomes, quickly acidifying the phagosomal lumen by inward pumping of protons, which initiates degradation of the cargo. The phagosome also acquires the **NADPH oxidase complex** (NOX), which produces reactive oxygen species (ROS). During phagolysosomal fusion, the organelle acquires hydrolytic enzymes, including glycosidases, lipases, DNAses, and proteases, such as cathepsins, which require a low pH for optimal activity [10]. Phagosomes also interact with a range of other organelles, such as Golgi-derived vesicles, the endoplasmic reticulum (ER), and mitochondria [11]. The contribution of the ER to phagosomal membranes was part of a major controversy in the field. While early results obtained by biochemical and morphological approaches indicated ER recruitment to phagosomes [12], other studies did not detect a significant contribution of the ER to forming or maturing phagosomes [13]. More recent data obtained by quantitative proteomic approaches suggest that the ER is part of the phagosomal membrane contributing approximately 20% of the early phagosome proteome in MΦs [11]. This study also revealed that only a subset of ER proteins is recruited to the phagosome, indicating that specific ER subdomains might contribute to this process.

While phagosome maturation is generally conserved, its extent and outcome are dependent on the phagocyte type. Phagosomes in neutrophils and in most MΦ populations rapidly destroy phagocytic cargo. By contrast, DC phagosomes often only partially degrade their cargo to preserve antigenic peptides for presentation to T lymphocytes to initiate adaptive immune responses. Hence, phagosome maturation in phagocytes is adapted to their specific function in immunity [14].

Recently, increasing evidence indicates that the phagosome is more than a degradation machinery and also functions as a signaling platform [15]. The main question we address here is how receptors, co-receptors, cytokines, and other immune signals influence phagosome maturation and its functional outcome: pathogen killing during an infection and antigen presentation for the activation of adaptive immunity. Recent proteomic studies shed light on the influence of immune stimuli on the phagosomal proteome and its functions 16, 17. Due to space constraints, we limit the scope of this review to MΦs and DCs, but refer the reader to excellent reviews describing mechanisms of phagocytosis in neutrophils and nonprofessional phagocytes 18, 19.

## Characteristics of Phagosome Maturation in MΦs and DCs

As mentioned above, kinetics of phagosome maturation display remarkable differences between MΦs and DCs. Phagosome maturation proceeds quickly in most MΦs, as illustrated by the rapid acquisition of hydrolytic enzymes and the v-ATPase [10]. MΦ phagosomes acidify strongly and rapidly, which inhibits microbial growth and activates many proteases present in the phagosome, such as cathepsin L [10]. Fast maturation kinetics are important for MΦ functioning, because rapid degradation of phagocytosed dead cells prevents the presentation of self peptides to T cells and the development of autoimmune diseases. Furthermore, fast maturation kinetics enable rapid killing of internalized pathogens, but hamper efficient presentation of pathogenic peptides. Nonetheless, this notion depends on the investigated tissue, because many MΦ subsets are able to present phagocytosed antigens to T cells efficiently and to initiate adaptive immunity. This is illustrated by the finding that both injected MΦs and DCs can migrate from blood and peripheral tissues into lymphoid organs to prime CD8^+^ T cell responses [20].

In contrast to most MΦs, DCs are believed to only partially degrade phagocytosed pathogens to preserve peptides for presentation to T cells. Consequently, acidification and antigen degradation kinetics of DC phagosomes are slower compared with MΦs [21]. Reduced acidification is a result of low v-ATPase and high NOX activity [22]. Moreover, DC phagosomes contain protease inhibitors and reduced levels of proteases [23] contributing additionally to the partial degradation of cargo. Other findings suggest that phagosomal proteolysis is facilitated by NOX independent of pH changes via luminal redox modulation of cysteine cathepsins [24], which influences the pattern of the MHC repertoire [25]. In any case, the resulting antigenic peptides from phagocytosed pathogens are presented either on MHC II molecules to CD4^+^ T cells or on MHC I molecules to CD8^+^ T cells [14].

## Impact of Immune Signals on Phagosome Maturation

Recently, various factors were identified that have direct or indirect impacts on phagosome maturation. These immune stimuli are present in the cell environment, such as **pathogen-associated molecular patterns** (PAMPs), **damage-associated molecular patterns** (DAMPs), and cytokines, and can be sensed at the cell membrane, or are present on the phagocytosed particles themselves, such as PAMPs, DAMPs, and **opsonins**. The effect of a specific stimulus, such as the one of lipopolysaccharide (LPS) of Gram-negative bacteria, may differ depending on whether it is present in the phagocyte environment or at the phagocytosed particle. Immune signals may accelerate or delay phagosome maturation to enhance microbial killing or antigen presentation, respectively. Below, we first discuss the impact of different immune signals present in the cellular or tissue environment, and then focus on the signals present at phagocytosed particles and their effects on phagosome maturation (summarized in Table 1).

**Table 1:** Impact of Immune Signals on Phagosome Maturation and Antigen Presentation^a^

Signal	Duration of stimulation	Type of phagocyte	Effect on phagosome maturation	Effect on Antigen Presentation	Phagocytic Cargo	Refs
Environmental immune stimulus						
PAMPs						
LPS (TLR4)	0–6 h	DC	No effect	No effect on XP	OVA polystyrene beads	[33]
	22 h; 7–20 h	DC	Delay	Enhanced XP	OVA polystyrene beads, apoptotic HSV-infected HeLa cells, pHrodo^®^*Escherichia coli*	33, 36
	20–40 h	DC	Delay	No effect on XP	OVA polystyrene beads	[33]
	18–22 h	MΦ	Delay	ND	Mannosylated silica beads	[34]
polyU (TLR7)	During uptake	DC	Delay	Enhanced XP	OVA polystyrene beads	[35]
R848 (TLR7/8)	16 h	DC	Delay	ND	OVA polystyrene beads	[33]
CpG (TLR9)	16 h	DC	Delay	ND	OVA polystyrene beads	[33]
	22 h	DC	No effect	Impaired XP, impaired CIIP	OVA polystyrene beads, apoptotic HSV-infected HeLa cells, pHrodo^®^*E. coli*	[36]
Cytokines						
IFN-γ	16–20 h; 18–22 h; 24 h	MΦ	Delay	ND	IgG polystyrene beads, DQ-BSA beads; mannosylated silica beads; polystyrene beads	34, 37, 38
	72 h + 48 h Mtb/CpG/LPS	MΦ	Delay	Impaired XP, impaired CIIP	OVA polystyrene beads	[41]
LPS + IFN-γ	18–22 h; 48 h	MΦ	Delay	ND	Mannosylated silica beads; serum-opsonized zymosan	34, 40
IL-4	1 h	MΦ	Delay	ND	IgG-opsonized zymosan	[44]
	48 h	MΦ	Acceleration	ND	IgG-opsonized silica beads; serum-opsonized zymosan	40, 43
IL-27	7 d	DC	Acceleration	Enhanced CIIP	Polystyrene beads, *Staphylococcus aureus*	[45]
TNF	22 h	DC	No effect	Impaired XP, no effect on CIIP	OVA polystyrene beads, apoptotic HSV-infected HeLa cells, pHrodo^®^*E. coli*	[36]
CD40 ligand	22 h	DC	No effect	No effect on XP, impaired CIIP	OVA polystyrene beads, apoptotic HSV-infected HeLa cells, pHrodo^®^*E. coli*	[36]
Particle-bound immune stimulus						
PAMPs						
Zymosan (TLR2 + Dectin1)		MΦ	Delay	Enhanced CIIP	Zymosan particles, *Candida albicans* beads, heat-killed *C. albicans*	[51]
PAM3CSK4 (TLR1/2)		MΦ	Acceleration	ND	Polystyrene beads	[49]
PAM3CSK4 (TLR1/2)		MΦ	No effect	ND	Polystyrene beads	[31]
TLR2/4 deficiency		MΦ	Delay	ND	Apoptotic cells, *E. coli, S. aureus*, *Salmonella typhimurium*	[29]
LPS (TLR4)		MΦ	Acceleration	ND	Avidin polystyrene beads	[16]
LPS (TLR4)		MΦ	No effect	ND	Polystyrene beads; OVA polystyrene beads	31, 50
LPS (TLR4)		DC	Acceleration	Enhanced CIIP	Apoptotic cells, OVA or EAP polystyrene beads, *E. coli and Saccharomyces cerevisiae*	30, 50
Trehalose dimycolate		MΦ	Delay	ND	BSA polystyrene beads	[47]
β-glucan (Dectin-1)		MΦ	Delay	ND	*C. albicans*; β-glucan beads	52, 67
β-glucan (Dectin-1)		MΦ	No effect	ND	Polystyrene beads	[53]
Mannan		MΦ	No effect	ND	Polystyrene beads; avidin polystyrene beads	16, 53
DAMPs						
Fibronectin		MΦ	No effect	ND	Polystyrene beads, C1q-opsonized *E. coli*	[53]
Calreticulin		MΦ	No effect	ND	Avidin polystyrene beads	[16]
Phosphatidylserine		MΦ	No effect	ND	Avidin polystyrene beads	[16]
Opsonins						
IgG		DC, MΦ	Acceleration	Enhanced XP, enhanced CIIP	*E. coli* expressing OVA (opsonized with deficient sera); OVA polystyrene beads	50, 54, 55, 56
IgG		MΦ	No effect	ND	Polystyrene beads; avidin polystyrene beads	16, 53
C1q		MΦ	No effect	ND	Polystyrene beads, C1q-opsonized *E. coli*	[53]
iC3b		MΦ	Acceleration	ND	iC3b-opsonized red blood cells; serum-coated *Yersinia pseudotuberculosis*, zymosan, *S. cerevisiae* and *E. coli*	[57]
iC3b		MΦ	No effect	ND	Polystyrene beads, C1q-opsonized *E. coli*; avidin beads	16, 53

a Abbreviations: CIIP, MHC II-restricted antigen presentation; ND, not determined; XP, MHC I-restricted cross-presentation of antigen; please see Glossary for further details.

### Effects of Environmental Stimuli

PAMPs are conserved molecular motifs associated with certain groups of pathogens that are recognized by **pattern recognition receptors** (PRRs). One family of PRRs are **Toll-like receptors** (TLRs), which recognize both intra- and extracellular microbial patterns and activate innate immune cells [26]. It has been proposed that TLR signaling is able to influence phagosome maturation in two ways, either globally during the activation of cellular TLRs or locally, when TLRs are present in phagosomes. Although the phagosomal localization of TLR2 and TLR4 has been shown 27, 28, their influence on phagosomal fate has been a matter of debate. Blander and Medzhitov showed that the maturation of phagosomes containing *Escherichia coli* but not those containing apoptotic cells was impaired in MyD88- and TLR2/TLR4-deficient MΦs compared with wild-type (WT) MΦs [29]. In WT cells, additional stimulation of TLR4 by LPS or simultaneous uptake of *E. coli* during phagocytosis of apoptotic cells did not influence phagolysosomal fusion kinetics of phagosomes containing apoptotic cells. The authors also demonstrated that, in DCs, the presence of TLR ligands within *E. coli* or apoptotic cell phagosomes promoted phagosomal antigen presentation to CD4^+^ T cells in a phagosome-autonomous way [30]. By contrast, when they analyzed kinetics of phagosome maturation and acidification by quantitative fluorometry techniques comparing particles with or without TLR ligands in WT and TLR2- or TLR4-deficient MΦs, Yates and Russell did not find evidence that TLR signaling affects phagosome maturation directly [31]. Instead, they argued that MΦ activation by TLR triggering affects phagosomal maturation. More recently, additional evidence indicated that TLRs present in phagosomes may be able to alter phagosome maturation (reviewed in [32]). However, more work is needed to identify unambiguously whether these findings are dependent on the analyzed physiological and pathological conditions.

**Figure 1:**
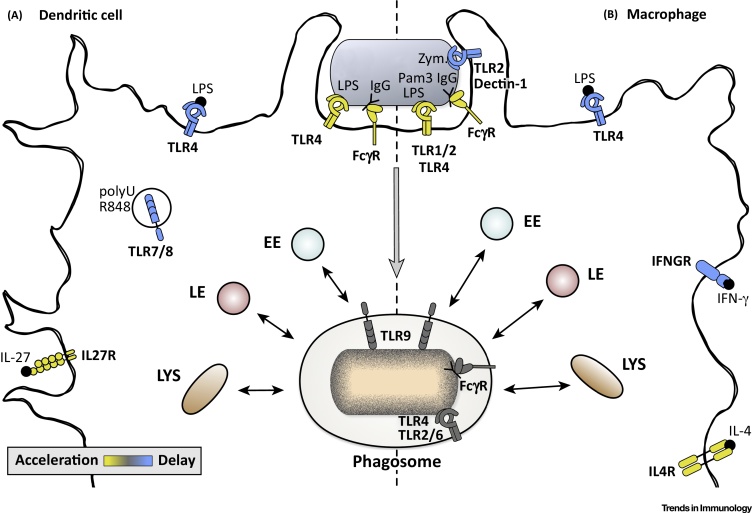
Key Figure: Impact of Immune Signals on Phagosome Maturation in Dendritic Cells (DCs) and Macrophages (MΦs)

In addition to their presence in phagosomes, TLR ligands can also be present in the extracellular environment (Figure 1), where they have different effects on phagosome maturation. One example is the TLR4 agonist LPS, whose effect on phagosome maturation kinetics is time dependent: short stimulation of DCs with LPS (up to 6 h) does not alter phagosomal antigen degradation, while intermediate (8–18 h) and long (20–40 h) stimulations induce a delay in phagosomal antigen degradation [33]. This is achieved by the perinuclear clustering of lysosomes, which is controlled by Rab34, leading to reduced phagolysosomal fusion. In turn, this results in the preservation of phagosomal antigenic peptides and efficient cross-presentation, an effect that is observed only transiently [33]. In MΦs, stimulation with LPS induced an M1-like phenotype with antitumor and antimicrobial activity and delayed phagosome maturation to enhance antigen presentation [34]. In DCs, stimulation of TLR7 by **polyuridylic acid** (polyU) also decreased phagosomal degradation and acidification *in vitro*, as measured by flow cytometry after incubation of cells with beads coupled to ovalbumin (OVA) or a pH-sensitive dye, respectively. Moreover, immunization of mice with polyU enhanced cross-presentation and promoted more efficient responses of cytotoxic T lymphocytes *in vivo* [35]. TLR9 stimulation with **CpG** only caused a minor delay in antigen degradation, yet increased phagosomal acidification and resulted in less efficient cross-presentation of phagosomal antigen in DCs 33, 36.

In addition to TLR agonists, cellular activation by cytokines can also directly influence phagosome maturation. Stimulation of MΦs with **interferon gamma** (IFN-γ) induced delayed phagosomal proteolysis, phagolysosomal fusion, and acquisition of maturation markers 34, 37, 38, 39. Comparison of the phagosomal proteome in resting and IFN-γ-stimulated MΦs identified proteins that are involved in phagosome maturation, microbe degradation, and cross-presentation 37, 39. IFN-γ delays phagosome maturation to promote the cross-presentation of phagosomal antigens by stimulating the recruitment of Rab20 to the phagosome, which prolongs association of Rab5a and phosphatidylinositol-3-phosphate (PI3P) to phagosomes [38]. Moreover, M1-like MΦ polarization by stimulation with IFN-γ and LPS for longer periods decreased not only phagolysosomal fusion, but also phagosomal acidification [34]. Decreased phagosomal acidification is caused by enhanced ROS production and reduced proton pump activity [40]. Similarly, co-stimulation with IFN-γ and CpG or with IFN-γ and *Mycobacterium tuberculosis* (Mtb) 19-kDa lipoprotein also delayed phagosome maturation [41]. By contrast, long-term stimulation of MΦs with IL-4 induced an M2-like phenotype. M2 MΦs are anti-inflammatory cells that promote antihelminth immunity and wound repair, and regulate metabolic homeostasis to efficiently clear dead cells [42]. M2-type MΦs display accelerated phagosome maturation kinetics, which may prevent the presentation of self-peptides and autoimmunity. Accordingly, phagosomal acidification is accelerated in these cells [40] and the proteolytic capacity of their phagosomes is enhanced [43]. By contrast, short-term stimulation of MΦs with IL-4 delayed the acquisition of phagosome maturation markers and phagosomal acidification after uptake of **immunoglobulin G** (IgG)-opsonized **zymosan** [44]. Moreover, stimulation of DCs with IL-27 for several days induced enhanced phagosomal acidification, as measured by increased co-localization of phagosomes with lysotracker, and enhanced acquisition of proteolytic enzymes, such as cathepsin D [45]. By contrast, stimulation of DCs with TNF and **CD40 ligand** did not induce major differences in phagosomal degradation [36].

In conclusion, although the effect of TLR signaling on phagosome maturation remains a controversial topic, under specific conditions TLR activation by stimuli in the cell environment can delay phagosome maturation to promote efficient antigen presentation. The extent of these effects depends on the specific TLR involved and the duration of stimulation. Similarly, M1-like MΦ polarization negatively regulates phagosome maturation kinetics, whereas M2-like MΦ polarization accelerates phagosome maturation. Yet, more research is needed to unravel the mechanistic details and to examine the effect of other immune stimuli, such as TGF-β, on phagosome maturation.

### Effects of Particle-Bound Stimuli

Not only immune stimuli present in the phagocyte environment, but also immune signals present at the phagocytosed particle itself can impact phagosomal maturation. Upon phagocytosis of microbes, their PAMPs can influence the kinetics of phagosome maturation. Since most microbes express a diverse variety of PAMPs, inert particles coated with a particular PAMP can be used to study the specific effects of this PAMP on phagosome maturation (Box 2). This model system can provide insight into the mechanisms underlying phagosome maturation, although it is important to verify findings in infection models using entire microbes as phagosomal cargo. For example, studies using beads coupled to mycobacterial glycolipids, such as lipoarabinomannans [46] or trehalose dimycolate [47], have shown that these ligands delay phagosome maturation and partially mimic the maturation block induced by live Mtb. Nonetheless, one has to be careful in the interpretation of these results, because single ligands sometimes do not reflect the physiological conditions initiated by a single bacterium [48].

Box 2 **The Latex Bead Model System**

During the 1960s, Wetzel and Korn developed an ingenious method using polystyrene particles to isolate latex bead-containing phagosomes (LBPs) from *Acanthamoeba castellanii* [98]. However, it was only during the early 1990s that this method was adapted to mammalian cells by the pioneering work of Desjardins and Griffiths to isolate phagosomes from murine MΦs [99]. Subsequently, it has been used by many labs, given that functionalized beads can be coupled to specific ligands and LBPs can be isolated at very high purity [100]. After phagocytic uptake, cells are mechanically disrupted to release LBPs, which are separated from other cell organelles and debris by density flotation on a discontinuous sucrose gradient 3, 98, 99. Remarkably, LBPs display most of the characteristics of microbe-containing phagosomes because they interact with the actin and microtubule cytoskeleton, and undergo fusion and fission events with vesicles of the endocytic compartment 101, 102, 103. As a result, LBPs are able to acidify, process, and load phagosomal antigens for presentation to T lymphocytes 104, 105, 106.

The use of this model system is sometimes more advantageous than the use of other phagocytic cargo, especially if one aims to isolate pure and homogeneous phagosome preparations for mass spectrometry. Due to the buoyancy of the latex beads, the isolation of LBPs requires only one to two centrifugation steps to separate the floating fraction containing the LBPs from contaminants trapped in other sucrose layers. By contrast, the isolation of bacteria-containing phagosomes is more laborious and yields less pure phagosome preparations 60, 107. Phagosomes also interact with autophagosomes [49]. Thus, remnants of organelles that are sent for degradation can be identified in LBP preparations. Since latex beads are nondegradable, they allow the isolation of LBPs at any stage of phagosome maturation [103], but cannot be used for studies on terminal cargo degradation and secretion of phagosomal content.

In addition to the mentioned advantages, functional assays in combination with flow cytometry are also available for accurately quantifying phagosome maturation and phagocytic uptake at a single organelle level 33, 50. Finally, latex beads coated with specific ligands facilitate the uptake of beads via a specific phagocytic receptor [100] and can be used to study one particular pathway at a time 16, 53.

Alt-text: Box 2

In MΦs, coating of beads with the lipopeptide **Pam3CSK4**, which is recognized by the TLR2/TLR1 heterodimer, enhanced phagosomal acidification [49]. Similarly, LPS-coated beads showed an increase in phagosomal acidification and oxidative burst, yet no major difference was observed in phagosomal antigen degradation 16, 50. In DCs, where phagosome maturation occurs less rapidly, LPS-coated beads exhibited increased phagosomal degradation and acquisition of the maturation marker LAMP-1 [50]. By contrast, beads coated with the TLR2 and Dectin-1 agonist zymosan stalled the acquisition of the phagosome maturation markers Rab7 and LAMP-2 and delayed phagosomal acidification of LC3-positive phagosomes by upregulating ROS production [51]. The recruitment of the autophagy protein LC3 to phagosomes characterizes a form of phagocytosis known as ‘LC3-associated phagocytosis’ (LAP) (Box 3). In agreement with the findings using zymosan particles, a study using β-glucan-coupled beads, which are also recognized by Dectin-1, similarly delayed phagosomal maturation, demonstrating the dependence of phagosome maturation on receptor ligation and Syk kinase activation [52].

Box 3 **LC3-Associated Phagocytosis**

LC3-associated phagocytosis (LAP) is a form of phagocytosis that is characterized by the receptor-mediated recruitment of autophagy proteins, such as LC3, to the phagosome. Although LAP uses parts of the autophagy machinery, LC3-positive phagosomes are distinct from autophagosomes. The former contain extracellular instead of intracellular material and are surrounded by a single lipid membrane structure instead of a double lipid membrane [108].

LAP is triggered by phagocytic baits that contain PRR ligands, such as bacteria or beads coupled to zymosan, LPS, or Pam3CSK4 [49]. In addition, FcγR signaling by IgG [109], recognition of phosphatidylserine on apoptotic cells by TIM4 [110], and Dectin1 signaling by beads coupled to β-glucans and fungi 111, 112 also trigger the recruitment of LC3 to the phagosome. In addition, LC3 is also present on carboxylated latex-bead phagosomes [113], suggesting that LC3 recruitment is common to many phagosomes.

LAP is at the interface between phagocytosis and autophagy, using both autophagy proteins, such as the class III PI3-kinase Vps34, and endosomal, LAP-specific proteins, such as Rubicon. Rubicon promotes PI3P production by Vps34 and ROS production by NOX2, which are both essential for LAP 109, 112. However, not all autophagy proteins are essential for the progression of LAP, such as the preinitiation complex [112]. In this study, Atg5 and Atg7 were shown to be required for LAP induced by zymosan or *Aspergillus fumigatus*. However, another study showed that Atg5 and Atg7 were dispensable for FcγR-induced LAP in murine cells [114].

In most publications, LAP is induced by TLR-activating particles and pathogens, which induce MΦ activation and, thus, significantly alter phagosome functions. This might explain apparently contradicting results in murine cells, where LC3-positive phagosomes, formed after LAP, show accelerated phagosome maturation and cargo degradation [49], while in human phagocytes, LAP delays phagosome maturation to promote efficient antigen presentation on MHC II [51].

Strong evidence points toward an important role of LAP in immunity because it is involved in the regulation of IFN production [115] and clearance of dead cells [110]. Defects in LAP-associated proteins impair the efficient engulfment and digestion of apoptotic cells, thereby contributing to SLE pathogenesis [116]. Considering the potential role of phagosomal LC3 recruitment in human disease, more work will be needed to understand the contribution and regulation of LAP in pathological settings. In the coming years, the community will need to focus on how LC3 and other LAP-associated molecules are recruited to phagosomes and to clarify their exact contribution to phagosome maturation.

Alt-text: Box 3

DAMPs, which are associated with tissue damage and cell death, do not appear to influence phagosome maturation kinetics in MΦs, as demonstrated for **fibronectin**, **calreticulin**, or **phosphatidylserine** 16, 53. Coupling of fibronectin and calreticulin to beads did not influence the co-localization of phagosomes with the v-ATPase, while bead coupling of phosphatidylserine did not result in changes in phagosomal pH, proteolysis, and oxidative burst. By contrast, coating of beads with opsonins, such as complement or immunoglobulins, mimics opsonization of pathogens by the immune system to enhance their phagocytic uptake 16, 53. Although no major difference on phagosomal antigen degradation was found in these studies, other results suggest that IgG opsonization is also able to accelerate antigen degradation of MΦ and DC phagosomes [50]. In agreement with these findings, opsonization of *E. coli* with IgG increased phagosome maturation kinetics and phagosomal killing 54, 55, 56. The opsonization of red blood cells, zymosan, and different microbes with the complement factor iC3b was shown to accelerate phagosome maturation by actin tail formation at the phagosome [57]. Other studies demonstrated that coating of beads with C1q and iC3b did not influence phagosome maturation in MΦs, while opsonization of *E. coli* with C1q negatively influenced bacterial killing 16, 53.

In conclusion, only certain PAMPs and opsonins signal from within the phagosome to positively or negatively modulate phagosome maturation. Why other PAMPs and opsonins fail to influence phagosome maturation kinetics remains to be elucidated. Surprisingly, the tested DAMPs did not modulate phagosome maturation when coupled to beads. Therefore, in future studies, the use of more physiological cargo will be needed to study in detail the influence of DAMPs on phagosome maturation.

## Phagosome Maturation in the Context of Cellular Signaling and Autoimmunity

Initially, the role of phagosomes in innate immunity was seen as a function of a degradation machinery modulated by signaling events at the cell surface and within the cytosol. Over the past few years, more evidence has emerged demonstrating that the phagosome is a signaling platform that integrates intraphagosomal, intracellular, and extracellular signals. There is also evidence that signaling from the plasma membrane and the phagosome might be different, because plasma membrane-localized TLRs induce specific signaling from endocytic compartments [58]. For example, it was demonstrated that inhibition of endolysosomal fusion using the v-ATPase inhibitor bafilomycin diminishes type I IFN production upon TLR2 or TLR4 activation, but did not have any effect on proinflammatory responses [59], suggesting the existence of specific phagosomal signaling networks in innate immunity.

Additional insight came from the mass spectrometric analysis of phagosomal proteomes (Box 4), which are dynamic and change their composition substantially during phagosome maturation [60]. In a recent study comparing the proteomes of MΦ phagosomes containing particles conjugated to various ligands, the authors showed that the mTOR complex is differently recruited depending on the cargo [16]. Moreover, recent deep proteomics data of MΦ phagosomes identified a phagosomal recruitment of 61 protein kinases and 96 receptors, including well-characterized innate immune signaling platforms, such as RIP kinases and tyrosine kinases of the Src family. Likewise, proteins involved in inflammatory signaling pathways were identified in phagosomes of DCs and IFN-γ stimulated MΦs 37, 61. Although the presence of these proteins in the phagosome does not automatically mean that they are active in signaling, the identification of functional protein phosphorylation cascades on the phagosome indicates that receptors in the phagosomal membrane are still able to signal to the cytoplasm. Therefore, it is not surprising that over 1100 phosphoproteins were identified in the phagosomal proteome of MΦs, many of them involved in processes ranging from antigen presentation to autophagy, stress responses, and apoptosis [62]. Considering the role of protein phosphorylation in regulating almost all cellular processes, it is likely that even ‘hard-wired’ processes, such as phagosome maturation, are influenced by intracellular signaling cascades. The molecular regulation of vesicle trafficking processes through post-translational modifications and their effect on innate immunity is understudied and, thus, more work is needed in this area.

Box 4 **Phagosome Proteomics**

Through optimization, latex bead phagosomes [99] or bacterial vacuoles [69] can be isolated at high purity using density gradient ultracentrifugation. In recent years, this high purity allowed the thorough characterization of the protein composition of latex bead phagosomes 1, 16, 39 and some bacterial phagosomes, such as *Legionella* [75] and *Mycobacterium* [117] vacuoles, by mass spectrometry-based proteomics. For this technique, phagosomal proteins are extracted, digested into peptides, and analyzed by liquid chromatography tandem mass spectrometry (LC-MS/MS), which provides both the identity of the proteins and their abundance.

Since phagosomes are dynamic intracellular organelles, their composition changes significantly during maturation and in response to external stimuli and the cellular activation status 16, 39. Being a target for various vesicle trafficking pathways, they can be used as a tool to characterize defects in specific arms of the endolysosomal pathway (Hartlova *et al.*, unpublished data, 2017). Phagosomal proteomes are relatively small (∼3000–4000 proteins) compared with the cellular proteome, but have wide ranges in protein abundance, with an estimated 40 proteins comprising approximately 50% of the protein mass [113]. Nonetheless, deep analysis can be achieved by single MS runs from as little as 2 μg of sample [113]. Latex bead phagosome proteomics was essential in identifying key concepts of innate immunity and vesicle trafficking. For example, it helped to characterize components of the endolysosomal trafficking machinery [99], identified the fusion of phagosomes with the ER 11, 12, 118, which explained the longstanding mystery of antigen cross-presentation by MHC class I molecules 106, 119, and characterized the recruitment of many signaling pathways to phagosomes of proinflammatory MΦs [39].

Alt-text: Box 4

However, although phagosome maturation is modulated by cellular signaling, it is also able to affect signaling in return. One example comprises nucleic acid-sensing TLRs that are confined to endocytic compartments and that only become fully functional after proteolytic processing and cleavage of their ectodomains by endolysosomal proteases acquired by phagosomes during maturation. For example, in addition to different cathepsins, asparaginyl endopeptidase (AEP) has been identified as the key protease that controls the proteolytic maturation of TLR9 [63]. In DCs, stimulation with CpG enhances AEP activity, increasing the acidification of these compartments and, thus, indirectly stimulating the cleavage of TLR9. Moreover, some TLR9 mutants, which do not need proteolytic processing to activate signaling, can no longer discriminate between foreign and self DNA and lead to systemic inflammation in mice [64]. Due to space constraints, we cannot discuss additional examples, but they are summarized elsewhere [65]. These findings show that phagosome maturation is needed for certain receptors to become competent for signaling and demonstrate how this process is able to affect cellular signaling events.

Intracellular segregation of nucleic acid-sensing TLRs to endosomes is also thought to provide one level of protection from autoimmunity, which might occur when cell surface TLRs bind to self nucleic acids that are released during necrotic and apoptotic cell death. Defects in the engulfment and digestion of apoptotic cells lead to the release of intracellular molecules, such as DNA and histones, which induce inflammation and autoimmunity. Alternatively, autoimmune responses may be provoked after microbial infections. Recently, a new mechanism was identified whereby phagocytosis of infected apoptotic cells by DCs leads to the presentation of both pathogenic peptides and self peptides, inducing autoreactive Th17 cells and autoimmunity [66]. Thus, degradation and presentation of apoptotic cells and signaling from the phagosome are critical in the development of autoimmunity. For more information on apoptotic cell clearance and autoimmunity, we refer readers to an excellent review [19]. Further comparative studies should focus on how apoptotic cells and bacteria are degraded. This is of clinical importance because it may lead to the identification of relevant therapeutic targets.

## Consequences on Microbial Elimination

Since phagosome maturation is essential for host defense, several pathogens have developed mechanisms to interfere with phagosome maturation, to escape the phagosomal lumen, or to survive within phagosomes. For example, some fungal pathogens have developed strategies to interfere with Rab dynamics to subvert phagosome maturation 67, 68. An arrest in phagosome maturation prevents microbial killing and degradation as well as the presentation of pathogenic peptides and the induction of adaptive immunity. Current progress in the isolation of pure, pathogen-containing vacuoles is boosting proteomic analysis of these organelles [69]. For a detailed overview of the different strategies of pathogens to interfere with host immunity, we refer to reviews elsewhere 70, 71. Below, we summarize findings of two bacterial pathogens that have evolved mechanisms to directly alter phagosome maturation.

**Figure 2:**
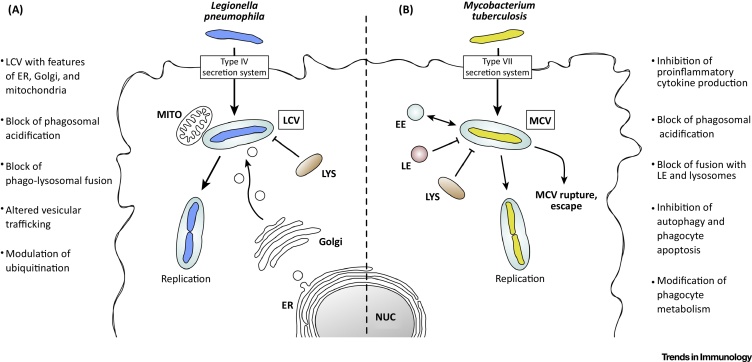
Strategies of Pathogens to Interfere with Phagosome Maturation and Host Immunity.. In this scheme, the major features that *Legionella pneumophila* (A) and *Mycobacterium tuberculosis* (B) have developed to evade host cell immunity are summarized. Their impact on phagosome maturation and other phagocyte functions is indicated. Abbreviations: EE, early endosome; ER, endoplasmic reticulum; LCV, *L. pneumophila*-containing vacuole; LE, late endosome; LYS, lysosome; MCV, *Mycobacterium tuberculosis*-containing vacuole; MITO, mitochondrion; NUC, nucleus.

*Legionella pneumophila* (Lpn) is the causative agent of Legionnaires disease, which infects alveolar MΦs upon inhalation of contaminated aerosols [72]. Lpn is contained in vacuoles (LCVs) and impedes phagolysosomal fusion and acidification by injecting multiple effector proteins into the host cell [73]. The block in phagosomal maturation permits Lpn replication in the phagosome [74] and its spread after lysis of the host cell. A recent study identified 2307 host proteins and 547 bacterial proteins in LCVs [75]. The mitochondrial protein Immune-responsive gene 1 (IRG1) was identified as a crucial regulator of immunity during Lpn infection. IRG1 expression is induced by type I and II IFNs, which impair the growth of Lpn by modulating host gene expression and the protein composition of LCVs. IRG1 is recruited to LCVs and mediates the production of the antimicrobial metabolite itaconic acid, which limits bacterial growth [75]. The different strategies that Lpn applies to interfere with host immunity are summarized in Figure 2 and are reviewed in [76].

Another pathogen that directly interferes with phagosome maturation is Mtb. Mtb causes 1.8 million deaths and over 10 million new cases of TB annually. It is transmitted via contaminated aerosols and infects mainly alveolar MΦs, but also DCs and epithelial cells. After its engulfment, Mtb arrests phagosome maturation at an early stage, preventing acidification, phagolysosomal fusion, and acquisition of lysosomal hydrolases 77, 78, 79 (Figure 2). More recently, it was also shown that Mtb is able to escape the phagosome and replicate within the cytosol [48]. Although Mtb induces a phagosome maturation arrest, certain immune stimuli can overwrite this block, promoting elimination of this pathogen. For example, IL-10 deficiency in MΦs promoted the acidification of MCVs [77]. In addition, M1-like stimulation of MΦs with IFN-γ triggered autophagy and, as a result, clearance of Mtb [80]. By contrast, M2-like polarization by IL-4 and IL-13 abolished mycobacterial control through autophagy [81]. More work is needed to better understand how the modulation of immune signals can help to induce phagosome maturation resulting in the killing of these pathogens. Therefore, detailed knowledge about the intimate relationship between pathogen secretion systems, translocated effectors proteins, and their impact on phagosome maturation will be valuable to better understand the virulence of Mtb and other pathogens.

## Concluding Remarks and Perspectives for Future Research

The dynamic and profound changes that phagosomes undergo during their maturation enable immune cells to maintain homeostasis and to respond rapidly to microbial threats. Therefore, phagosome maturation has a direct impact on the outcome of immune responses and is regulated not only at the cellular level, but also at the organelle level. In recent years, an increasing body of evidence has demonstrated several links between the phagosome maturation machinery and different signaling pathways. In addition, polarization of phagocyte cell populations enables the innate immune system to initiate and shape immune responses. For example, the M1-like and M2-like phenotypes during MΦ polarization are able to induce different features of phagosome maturation, even when they are located in the same tissue. Interestingly, these characteristics are not restricted to MΦs, as nicely demonstrated by the emerging concept of neutrophil polarization [82].

The kinetics of phagosomal maturation are susceptible to various stimuli ranging from cytokines and PAMPs to DAMPs and opsonins. Other factors, such as the duration of stimulation and the engaged phagocyte type, also influence phagosomal fate. Therefore, phagosome maturation can be stimulated to enhance pathogen killing or to prevent presentation of self peptides and autoimmunity. By contrast, phagosome maturation can be delayed to preserve pathogenic peptides for presentation to T cells to efficiently induce adaptive immunity. More work is needed to identify the signaling pathways to and from the phagosome and to better understand how different phagocytes regulate these aspects at the molecular level. It is currently not known whether immune signals modulate phagosome maturation upon triggering of a single signal transduction pathway or if multiple pathways are involved. In addition, the effects of other types of environmental stimuli (e.g., allergens) have not yet been studied (see Outstanding Questions).

Based on current progress, the idea emerges that phagosomes function as signaling platforms that integrate multiple intraphagosomal, intracellular, and extracellular signals, which each can modulate phagosome maturation. Proteomic studies revealed that many proteins known to be involved in signaling, such as receptors and kinases, are present in phagosomes. The phosphorylation status of these proteins is only one aspect that influences phagosome maturation. Finally, the various interactions between phagosomes, the inflammasome, and surrounding organelles, such as autophagosomes and mitochondria, suggest that the phagosome is not a solitary organelle and can communicate to other organelles. Future research could focus on these interactions and the impact of immune signals, especially when phagosome maturation is impaired by pathogens to evade host immunity. This might help to develop new therapeutics that enhance pathogen killing and adaptive immunity.

Outstanding Questions What are the molecular signaling pathways to and from the phagosome and how are they regulated?

Are immune signals able to modulate phagosome maturation upon triggering of a single signal transduction pathway or are multiple pathways involved? Is it possible to identify common key molecules?

Which Rab proteins have key roles in the modulation of phagosome maturation?

Are phagosomes only involved in intracellular communication or do they also participate in intercellular communication?

What is the effect of other types of environmental stimulus, such as allergens, on phagosome maturation?

How can the phagosome as a signaling platform be modulated by new therapeutics to enhance antigen presentation and pathogen killing?

## Glossary

**β-glucan**: polysaccharide in the cell wall of bacteria and fungi; recognized by Dectin-1 (Clec7A).
**Calreticulin**: signaling protein in the endoplasmic reticulum (ER); also exposed on the plasma membrane of apoptotic cells, initiating their clearance by immune cells.
**CD40 ligand**: ligand of the cell surface antigen receptor CD40; expressed on the plasma membrane of activated T cells.
**CpG**: short, single-stranded oligodeoxynucleotides that contain a cytosine triphosphate deoxynucleotide linked by a phosphodiester to a guanine triphosphate deoxynucleotide; they are PAMPs recognized by TLR9 in endosomes.
**Damage-associated molecular pattern (DAMP**): intracellular molecule that is released upon cell damage causing inflammation.
**Fibronectin**: glycoprotein of the extracellular matrix that binds to membrane-spanning integrins.
**Immunoglobulin G (IgG)**: antibody found in blood and extracellular fluid; controls infection of tissues by IgG-mediated coating of pathogen surfaces (opsonization), which facilitates recognition by Fcγ receptors and clearance by phagocytic immune cells.
**Inflammasome**: multiprotein complex of the innate immune system in myeloid cells; assembled during fungal, bacterial, and viral infections; activation induces maturation of proinflammatory cytokines (e.g., IL-1β and IL-18) and pyroptosis.
**Interferon gamma (IFN-γ)**: cytokine and type II interferon important in cell signaling; mainly produced by natural killer (NK) cells and different T cells; induces strong antiviral and antitumor immune responses.
**Mannan**: polysaccharide in the cell wall of fungi and bacteria; recognized by the mannose receptor.
**NADPH oxidase (NOX)**: enzyme complex in plasma membrane and phagosomal membranes; produces oxygen radicals to generate reactive oxygen species (ROS), which are important in innate immunity and pathogen killing.
**Opsonin**: molecule that enhances phagocytosis by binding and marking pathogens or dead cells for subsequent uptake by phagocytic immune cells (e.g., C1q and iC3b).
**Pam3CSK4**: synthetic triacylated lipopeptide that mimics the acylated terminus of bacterial lipopeptides; recognized by the TLR1/TLR2 heterodimer on the cell surface of immune cells.
**Pathogen-associated molecular pattern (PAMP)**: conserved motif associated with groups of pathogens that is recognized by PRRs.
**Pattern recognition receptor (PRR)**: essential component of innate immunity; comprises different receptor families that are able to recognize PAMPs and DAMPs.
**Phosphatidylserine**: phospholipid present in the inner leaflet of the plasma membrane, which is flipped to the outer leaflet upon apoptosis; recognized during the anti-inflammatory clearance of apoptotic cells.
**Polyuridylic acid (polyU)**: synthetic, single-stranded RNA that only contains uridine subunits; recognized by murine TLR7 and human TLR8 in endosomes of immune cells.
**R848**: synthetic guanosine derivative with potent antiviral activity; recognized by murine TLR7 and human TLR8 in endosomes of immune cells.
**Toll-like receptor (TLR)**: PRR family comprising 13 members in mammals; recognizes conserved viral, bacterial, and fungal PAMPs; present either on the cell surface and/or in endosomes and phagosomes of immune cells
**Vacuolar (v)-ATPase**: vacuolar proton pump utilizing energy of ATP hydrolysis to transport protons across intracellular and plasma membranes; acquired by phagosomes from endosomal and lysosomal compartments to acidify their lumen.
**Zymosan**: fungal β-glucan prepared from yeast cell wall; recognized by TLR2 and Dectin-1.
